# Adverse Impact of Diet-Induced Hypercholesterolemia on Cardiovascular Tissue Homeostasis in a Rabbit Model: Time-Dependent Changes in Cardiac Parameters

**DOI:** 10.3390/ijms140919086

**Published:** 2013-09-17

**Authors:** Attila Kertész, Mariann Bombicz, Daniel Priksz, Jozsef Balla, Gyorgy Balla, Rudolf Gesztelyi, Balazs Varga, David D. Haines, Arpad Tosaki, Bela Juhasz

**Affiliations:** 1 Department of Cardiology, Faculty of Medicine, University of Debrecen, Nagyerdei krt. 98, Debrecen 4032, Hungary; E-Mail: dr.kertesz.attila@gmail.com; 2 Department of Pharmacology, Faculty of Pharmacy, University of Debrecen, Nagyerdei krt. 98, Debrecen 4032, Hungary; E-Mails: bombicz.mariann@pharm.unideb.hu (M.B.); priksz.daniel@pharm.unideb.hu (D.P.); gesztelyi.rudolf@pharm.unideb.hu (R.G.); varga.balazs@pharm.unideb.hu (B.V.); david.haines@uconn.edu (D.D.H.); arpad.tosaki@pharm.unideb.hu (A.T.); 3 MTA-DE Vascular Biology, Thrombosis and Hemostasis Research Group, Hungarian Academy of Sciences, University of Debrecen, Nagyerdei krt. 98, Debrecen 4032, Hungary; E-Mails: balla@internal.med.unideb.hu (J.B.); balla@med.unideb.hu (G.B.); 4 Department of Nephrology, Medical and Health Science Center, University of Debrecen, Nagyerdei krt. 98, Debrecen 4032, Hungary; 5 Department of Pediatrics, University of Debrecen, Nagyerdei krt. 98, Debrecen 4032, Hungary

**Keywords:** hypercholesterolemic rabbit, cardiac parameters, echocardiography, heme-oxygenase, cytochrome oxidase, VEGF

## Abstract

The present study evaluates a hypothesis that diet-related hypercholesterolemia increases oxidative stress-related burden to cardiovascular tissue, resulting in progressively increased mortality, along with deterioration of electrophysiological and enzymatic function in rabbit myocardium. New Zealand white rabbits were divided into four groups, defined as follows: GROUP I, cholesterol-free rabbit chow for 12 weeks; GROUP II, cholesterol-free chow, 40 weeks; GROUP III, chow supplemented with 2% cholesterol, 12 weeks; GROUP IV, chow supplemented with 2% cholesterol, 40 weeks. At the 12 and 40 weeks time points, animals in each of the aforementioned cohorts were subjected to echocardiographic measurements, followed by sacrifice. Significant deterioration in major outcome variables measured in the present study were observed only in animals maintained for 40 weeks on 2% cholesterol-supplemented chow, with much lesser adverse effects noted in animals fed high cholesterol diets for only 12 weeks. It was observed that rabbits receiving high cholesterol diets for 40 weeks exhibited significantly increased mortality, worsened ejection fraction and general deterioration of cardiac functions, along with increased atherosclerotic plaque formation and infarct size. Additionally, myocardium of GROUP IV animals was observed to contain lower levels of heme oxygenase-1 (HO-1) and cytochrome *c* oxidase III (COX III) protein relative to the controls.

## 1. Introduction

Cardiovascular diseases remain the major cause of morbidity and mortality in industrialized Western nations. In these disorders, death most often occurs as a consequence of atherosclerosis, leading to stroke, ischemia, myocardial infarction, heart failure and other syndromes characterized by severely dysregulated inflammatory processes and their resulting degradation of tissue function [1,2]. A hallmark of these syndromes is microvascular damage associated with a progressive decline in healthy cardiovascular tissue homeostasis, which typically is accompanied by diminished arterial flexibility and onset of age-associated pathologies, especially cardiovascular, neurologic and kidney disease [3]. The association of dyslipidemia and ischemic heart diseases are well known. Nevertheless, at the time of this writing, a detailed description of the etiology and molecular pathomechanisms of hypercholesterolemia-induced atherosclerosis and its complications remains to be determined [3].

Recent evidence suggests that mitochondria are particularly sensitive to hypercholesterolemia-associated oxidative stress; therefore, the morphological and functional analysis of mitochondria is expected to contribute to the prevention of and therapy for hypercholesterolemia-induced diseases [4].

Hypercholesterolemia is associated with elevated levels of malondialdehyde (MDA), a measure of oxidative stress in rabbit aorta [5–8]. Hypercholesterolemia is also known to increase the production of reactive oxygen species (ROS) through various pathomechanisms. Mitochondrial electron-transfer complexes are major sources of ROS, and oxidative damage to the electron transport complexes that are in close proximity to these ROS sources, e.g., cytochrome *c* oxidase, are expected to inhibit electron transport. Such inhibition increases electron leakage, leading to further ROS production [9]. The resulting intensely oxidative environment may inactivate cytochrome *c* oxidase, resulting in tissue damage—an example being doxorubicin-induced cardiomyopathy [10].

Physiologically relevant pathological changes observed as a result of long-term hypercholesterolemia may also occur as a result of brief hypercholesterolemic episodes (days), with profound adverse effects on endothelium-dependent functions of the microcirculation, including dilation of arterioles, fluid filtration across capillaries and regulation of leukocyte recruitment in postcapillary venules [11]. A further consequence of hypercholesterolemia-induced microvascular responses may include enhanced vulnerability of the microvasculature to the harmful effects of ischemia and other inflammatory conditions [11]. Moreover, increased sensitivity to ventricular fibrillation exhibited by the hypercholesterolemic heart is also observed and is associated with prolonged action potential duration, longer QT (measure of the time between the start of the Q wave and the end of the T wave in the heart's electrical cycle) intervals and increased repolarization dispersion [12].

After atherosclerotic-related etiologies, heart failure (HF) is the second major contributor to the incidence of cardiovascular dysfunctions [13]. HF is also a leading cause of death in middle- and high-income nations [1]. Heart failure is not classed as a distinct disease, but generally considered to be a group of symptoms, which may be triggered or exacerbated by a major atherosclerotic condition, hypertension, valvular diseases, cardiomyopathy and adverse drug effects [1]. Congestive heart failure manifests as left- or right-sided or biventricular, and the known major risk factors include obesity, hypertension, smoking and diabetes [14]. Currently available therapies may slow the progression of this pathology, but not cure it [15]. Moreover, right-sided heart failure is considered to be incurable, and the disorder is refractory to most drugs [15].

The overall grim outlook for definitive treatment of cardiovascular disorders is nevertheless mitigated somewhat by the results of treatment modalities extracted from traditional and herbal medicine. Previous studies have demonstrated the significant cardiovascular benefits of a wide range of plant materials, including commonly used components of the human diet, as well as natural health products and nutraceuticals [16,17]. Previously, the authors demonstrated that sour cherry seed extract, a natural product that contains a powerful inducer of heme oxygense-1, strongly inhibits ischemia-reperfusion (IR) injury [3,18,19].

Strategies that augment the currently used pharmacotherapies offer the advantage of low-to-negligible toxicity, widespread availability and low price. For the aforementioned reasons, characterization of the biological effects mediated by herbal products opens opportunities in therapy for cardiovascular disorders.

The present investigation is expected to yield insight into mechanisms by which hypercholesterolemia-related oxidative stress degrades the function of critical macromolecules and promotes disease. Hypercholesterolemia is a well-established risk factor for coronary artery disease and is related to endothelial dysfunction at both the macro- and micro-vascular level. A significant finding of the present study is that the ejection fraction also was greatly diminished in animals maintained long-term (40 weeks) on high dose cholesterol diet (2%), with subsequent development of myocardial infarction.

The core hypothesis of the present investigation is that oxidative stress induced in a hypercholesterolemic rabbit model by sustained cholesterol diets promotes pathological changes in ways that may be revealed by changes on the molecular level. The investigators examined the effects of hypercholesterolemia on cardiac systolic and diastolic parameters using echocardiography. Mechanistic experiments were conducted that included measurement of the activity of cytoprotective enzymes, such as heme-oxygenase (HO-1), influences on revascularization of vascular endothelial growth factor (VEGF) protein, antiapoptotic influences and the effects of dietary treatments on mitochondrial function. Corollary studies also included assessment of the role of cytochrome *c* oxidase (COX) enzymes in regulating ATP metabolism and hosting adaptive, anti-inflammatory processes, including ROS scavenging.

## 2. Results and Discussion

**Figure 1:**
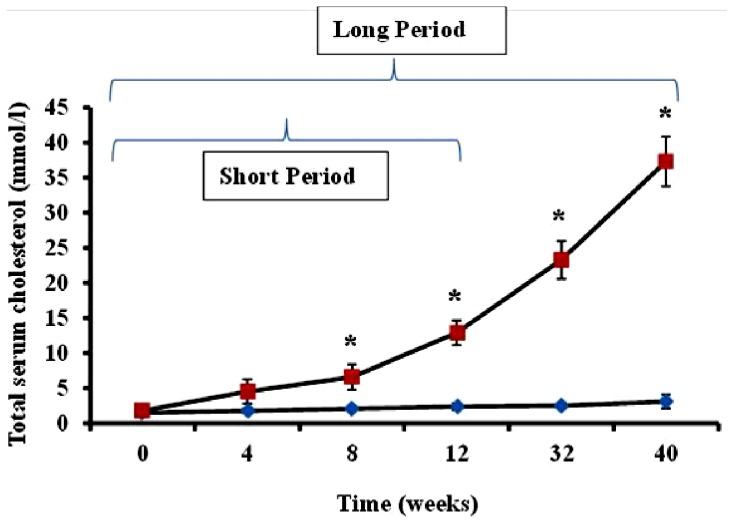
Time-dependent serum cholesterol level in normal and hypercholesterolemic rabbits. Average total serum cholesterol levels (mmol/L ± SEM) in two groups of rabbits (*n =* 4 per group), each administered feed containing 2% cholesterol, were measured using the CardioCheck serum cholesterol analyzer during a 12-week (short period) and 40-week (long period) time-course in hypercholesterolemic rabbits (squares) and non-hypercholesterolemic control animals (diamonds). *p < 0.05 for comparison of serum cholesterol in hypercholesterolemic *versus* non-hypercholesterolemic rabbits.

Serum cholesterol levels in animals administered feed supplemented with 2% cholesterol (hypercholesterolemic rabbits) and rabbits receiving normal feed (controls) were evaluated during the study period. As shown in Figure 1, total serum cholesterol in hypercholesterolemic animals provided with feed containing 2% cholesterol was significantly increased during the 40-week time-course of the study relative to levels observed in control rabbits receiving normal feed (*p <* 0.05).

Although hepatic functions were not specifically evaluated in the present study, the known consequences of hypercholesterolemia on the liver must be considered in the context of the influence of this organ on cardiac health. Sustained elevation in dietary cholesterol levels places a burden on the liver that contributes significantly to vascular deterioration [20]. Disruption of normal hepatic activity may adversely affect cardiac health through numerous pathways. One example of the interaction between these two organ systems, which may emerge as an area for development of novel therapies, is that the disruption of normal regulation by the liver of platelet-activating factor acetyl-hydrolase may strongly contribute to atherosclerosis, since this enzyme is low density lipoprotein (LDL)-associated [21]. It is therefore likely that other effectors of cardiovascular metabolism that are regulated by the liver and are high density lipoprotein (HDL)- and/or LDL-associated may contribute to cardiac pathologies as a result of hyperlipidemia.

**Figure 2:**
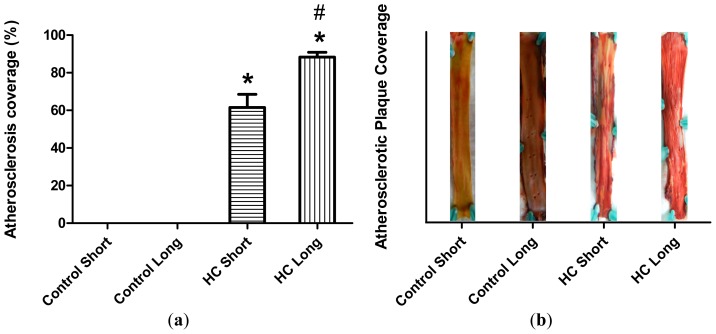
Arterial plaque coverage. (**a**) Atherosclerotic plaque coverage in Sudan III-stained luminal sections of thoracic arteries harvested from four groups of rabbits (*n* = 4 per group), administered normal feed (control) or feed supplemented with 2% cholesterol (HC, high cholesterol). Plaque coverage evaluated following a 12-week (short) or 40-week (long) time-course is shown as the average percentage of plaque coverage in arteries from each group of animals ± SEM; (**b**) The extent of atherosclerosis in arterial lumens is represented in histogram form as percentage plaque coverage; and as photographic images of representative arterial sections shown in the same frames as each histogram. *p < 0.05 for comparison of percentage atherosclerotic plaque coverage in arteries of non-hypercholesterolemic long (control long) rabbits *versus* hypercholesterolemic long (HC long) and hypercholesterolemic short (HC short) rabbits. # *p <* 0.05 for comparison of percentage atherosclerotic plaque coverage in arteries of hypercholesterolemic long (HC long) rabbits *versus* hypercholesterolemic short (HC short) rabbits.

Plaque coverage in thoracic arteries is shown in Figure 2. The extent of plaque coverage in animals maintained on high cholesterol diets (HC) was significantly greater than rabbits fed with normal chow (control) (*p <* 0.05), in which negligible atherosclerotic plaque accumulation was noted, as shown in the Figure 2 photographs of dissected arteries. Moreover, as expected, animals fed high cholesterol diets for 40 weeks (HC long) exhibited significantly higher plaque coverage than rabbits receiving the 2% cholesterol-supplemented feed for 12-week periods (HC short) (*p <* 0.05).

**Figure 3:**
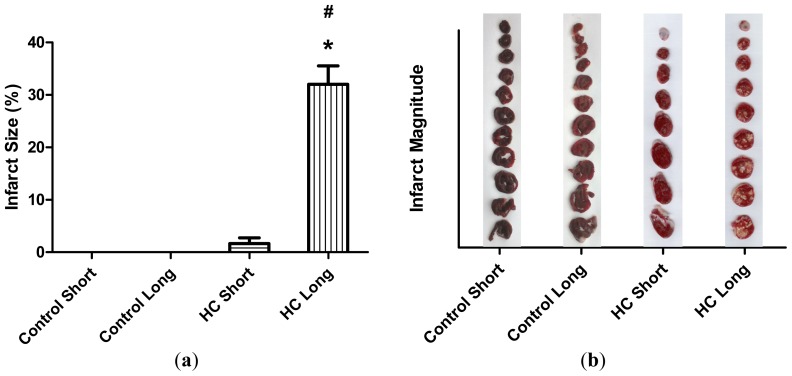
Total infarcted region. (**a**) Hearts were isolated from four groups of rabbits (*n* = 4 per group), administered normal chow for 12 weeks (control short); normal chow for 40 weeks (control long); high cholesterol (2%) chow during a 12-week time-course (HC short); or high cholesterol chow during a 40-week time-course (HC long). Isolated hearts were perfused in modified Krebs-Henseleit bicarbonate buffer using a Langendorff apparatus. The total infarcted region was determined by perfusion with triphenyl tetrazolium chloride (TTC) solution, followed by microscopic analysis of transverse sections of each heart. Average sizes of the infarct zone for hearts in each group ± SEM are shown for each treatment group; (**b**) The magnitude of infarct zones measure in hearts of animals subjected to selected treatment conditions is displayed in histogram form in the same frame spaces as photographs of infarcted heart sections. *p < 0.05 for comparison of average % infarct size in HC long *versus* HC short group. # *p <* 0.05 for comparison of average % infarct size in HC long *versus* control long group.

The average extent of infarct zones in control short, control long, HC short and HC long groups is represented by histograms in Figure 3. These outcomes reveal that infarcted regions did not develop in non-hypercholesterolemic (control) animals, with only small infarcted areas forming in hearts of rabbits receiving high cholesterol chow for 12-week periods (HC short). Conversely, infarcted regions were noted in hearts of rabbits fed with the 2% cholesterol-supplemented diet for 40 weeks (HC long), which were significantly larger than the infarct size of HC short animals (*p <* 0.05).

Hypercholesterolemia degrades homeostatic processes in cardiovascular tissue in ways that engender epicardial and microvascular coronary artery disease. Elevated levels of serum cholesterol promote formation of plaque deposits in epicardial arteries, which progressively narrow vessel lumen, depriving tissues they supply of oxygenated blood. As myocardial oxygen demand exceeds supply, infarct zones may develop in regions of myocardial tissue supplied by occluded blood vessels. In humans, the clinical presentation of such processes is termed “non-ST (connects the QRS complex and the T wave) elevation myocardial infarction”. The pathogenesis of this syndrome does not involve occlusion of the infarct-related coronary artery; therefore, no ST segment elevation—a sign of total coronary occlusion—is typically observed in electrocardiography (ECG) evaluations of afflicted individuals. For non-ST elevation myocardial infarction, the extent of necrotized tissue is dependent on the size of the myocardial region supplied by the coronary artery distal to the stenotic lesion and the time interval of the insufficient oxygen supply. Having severely stenotic coronary arteries and stained myocardial necrotic regions, the pathophysiology of myocardial infarction in HC long animals for the present study may be similar to non-ST segment myocardial infarction observed in humans. Furthermore, since the total infarcted region or myocardial mass increases, the systolic function of the heart is attenuated, causing a decrease in fractional shortening and the ejection fraction. Experimental ligation of the coronary artery creates disruption of tissue homeostasis that resembles ST segment elevation myocardial infarction observed in humans when the coronary artery is totally occluded by thrombus formation at the time of rupture of an atherosclerotic plaque. This infarcted region is clearly distinguishable from normal myocardial tissue and plain necrotic areas—and may, thus, be identified with little ambiguity, unlike in non-ST elevation cases.

**Figure 4:**
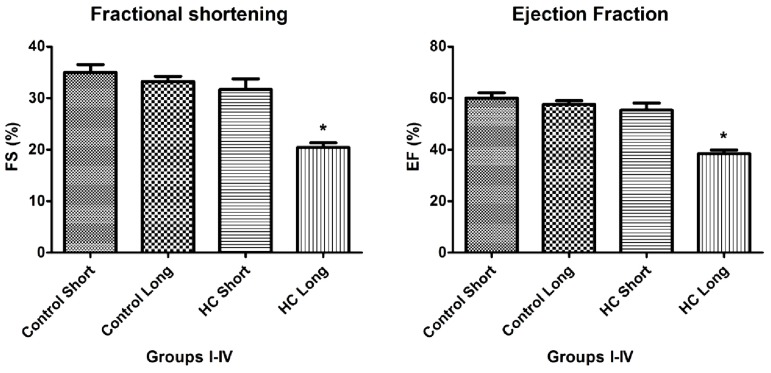
Fractional shortening (FS) and ejection fraction (EF) of left ventricles. Echocardiographic measurements were conducted on four groups of rabbits (*n* = 4 per group), administered normal chow for 12 weeks (control short); normal chow for 40 weeks (control long); high cholesterol (2%) chow during a 12-week time-course (HC short); or high cholesterol chow during a 40-week time-course (HC long). Measurements were made of fractional shortening (FS), determined as [left ventricle end diastolic diameter (LVEDD) – left ventricle end systolic diameter (LVESD)]/LVEDD, and ejection fraction (EF), calculated as (LVEDD^2^ − LVESD^2^)/LVIDD^2^, as shown. *p < 0.05 for comparison of average % FS or % EF with all other groups.

**Figure 5:**
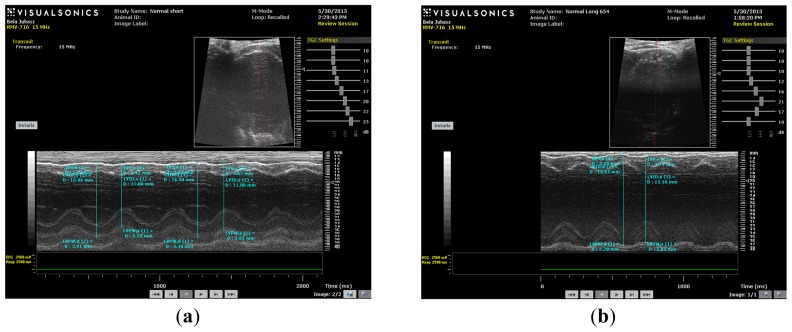

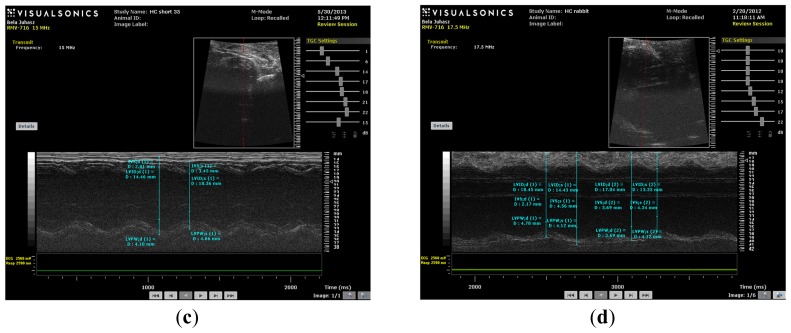
(**a**) M-mode image of control short (CS) heart; (**b**) M-mode image of control long (CL) heart; (**c**) M-mode image of HC short (HCS) heart; (**d**) M-mode image of HC long (HCL) heart.

As shown in Figures 4 and 5, the values of fractional shortening (FS) and ejection fraction (EF) of the left ventricle were significantly reduced in HC long animals relative to their values in non-hypercholesterolemia control rabbits (*p <* 0.05) and to animals maintained for shorter time periods on hypercholesterolemia-inducing chow (HC short) (*p <* 0.05). These outcomes are expected based on previous studies, which reveal significant positive correlation between the occurrence of cardiac insufficiency and associated heart failure with possible left ventricular hypertrophy [22–24]. The aforementioned echocardiographic data provides insight into the influence of high cholesterol on the underlying pathogenesis of cardiovascular disease and helps account for other observations described in the present report, such as increased infarct magnitude in hypercholesterolemia animals. No significant differences in Groups I–III were observed. This expectation shows that neither ageing itself nor short-term hypercholesterolemia afflict the systolic and diastolic parameters and normal heart function. Although, in animals receiving a long-term cholesterol diet, these values were significantly lower (*p <* 0.05), which shows that long-term hypercholesterolemia can deteriorate left ventricle systolic function. As seen in Table 1, no significant differences in other echocardiographic parameters were measured. It should be also noted that the left ventricle (LV) masses of Groups II and IV were higher than the LV masses of the short-treated animals, although this only represents that the LV mass is correlated with increasing body weight, since these animals were five months older at the moment of extermination.

M-mode images demonstrate cross-sections of the heart swept by the instrumentation over a defined time period. This evaluation is conducted by placing the cursor line on 2D images at the mid-ventricular level, with the largest diameter in either the parasternal long or parasternal short axis view (on the M-mode pictures at the upper part 2D views with the cursor; at the lower part, the tracing at the cursor line could be seen). This technique, which provides a very high temporal resolution, allows the end systolic [minimal diameters, left ventricular internal diameter at end-systole (LVIDs)] and end diastolic [maximal diameter, left ventricular internal diameter at end-diastole (LVIDd)] diameters to be precisely measured. These parameters are used to calculate fractional shortening [(LVIDd − LVIDs)/LVIDd] and ejection fraction [(LVIDd − LVIDs)^2^/LVIDd^2^] (cubed formula), which are indicators of left ventricular emptying capacity. Normally, the heart ejects more than 50% of its end diastolic volume (ejection fraction), and the diameter shortens more than 25% (linear ejection fraction or fractional shortening). When the myocardial tissue is damaged (typically by oxidative stress), its contractile function is attenuated and emptying capability decreased.

In the present study, hypercholesterolemia adversely affected the vasculature to result in severe atherosclerotic lesions of coronary arteries. The resulting reduction in the availability of blood and oxygen resulted in micro- and macro-myocardial infarctions (TTC perfusion sections—total infracted region). These multiple necrotic events caused global left ventricular contractility impartment that was detectable by decreased fractional shortening and ejection fraction parameters *in vivo* and by decreased aortic flow in isolated working heart specimens of HC animals.

**Figure 6:**
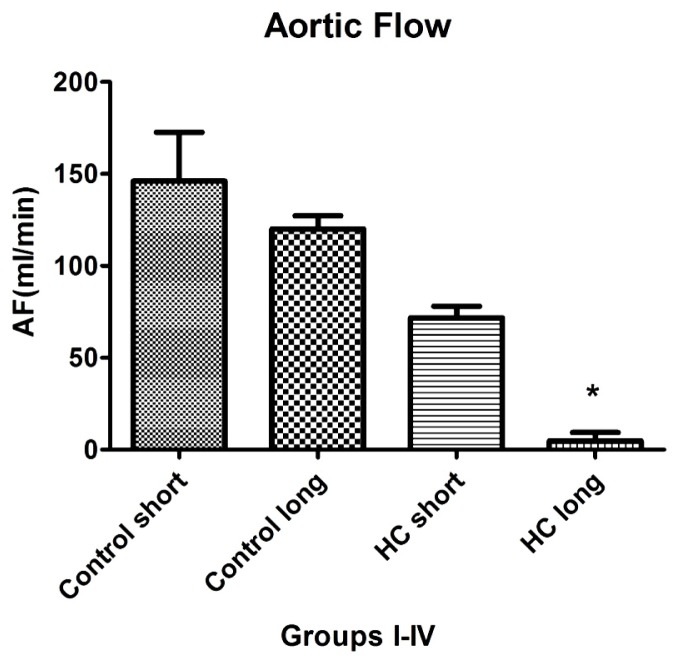
Aortic flow in isolated rabbit hearts. Aortic flow measured using a calibrated flow meter was determined in isolated working hearts from four groups of rabbits (*n* = 4 per group), administered normal chow for 12 weeks (control short); normal chow for 40 weeks (control long); high cholesterol (2%) chow during a 12-week time-course (HC short); or high cholesterol chow during a 40-week time-course (HC long). *p < 0.05 for comparison of average aortic flow in hearts from HC long animals with all other groups.

Aortic flow, the magnitude of which is a major indicator of cardiac health, was evaluated in isolated working hearts harvested from non-hypercholesterolemia rabbits (control) maintained on normal chow for 12 weeks (control short) or 40 weeks (control long). Aortic flow measurements were also made on hypercholesterolemia (HC) animals fed high cholesterol chow for 12-week periods (HC short) or 40 weeks (HC long). Figure 6 shows that HC long rabbits exhibited profoundly significant reduction in aortic flow relative to both non-hypercholesterolemia control groups and also with respect to rabbits fed 2% cholesterol for 12-week periods (HC short) (*p <* 0.05). After 12 and 40 weeks, rabbit hearts were subjected to isolated working heart examination. The perfusion medium consisted of a modified Krebs-Henseleit bicarbonate buffer. The left atrium was cannulated, and the Langendorff system was adapted and switched to isolated working hearts.

Induction of ischemia/reperfusion injury was not achieved in these experiments, since the hearts from hypercholesterolemic rabbits showed very poor heart functions (no recovery). Nevertheless, significant impairment of aortic flow in hypercholesterolemic rabbits compared to control animals was observed, along with a significant decrease in this parameter in the long-term cholesterol treated animals *versus* the short-term treated group (*p <* 0.05). Many animals failed to produce measurable aortic flow (0 mL/min); hypercholesterolemia resulting from a sustained high cholesterol diet can seriously degrade normal cardiac functions.

**Figure 7:**
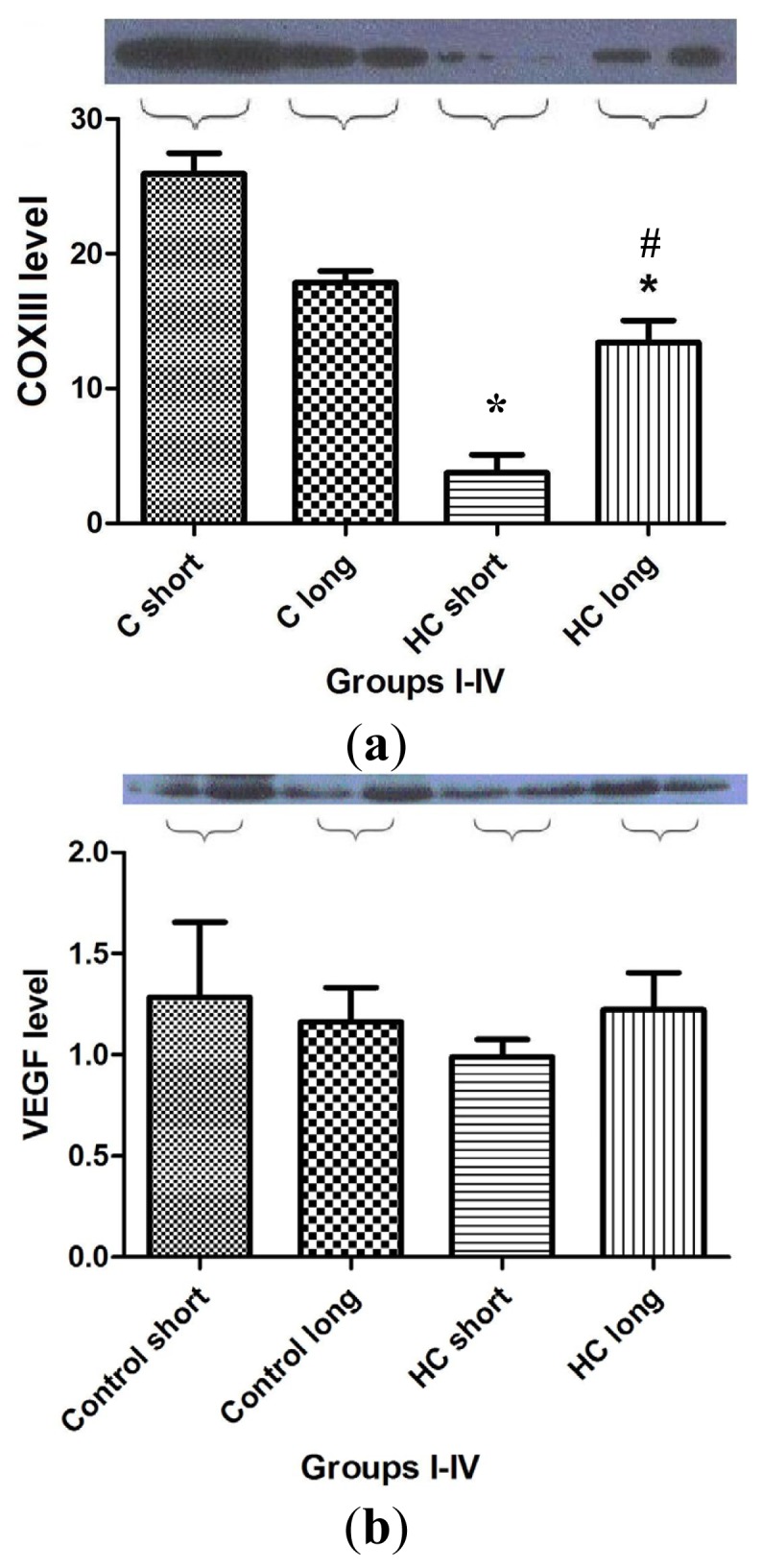

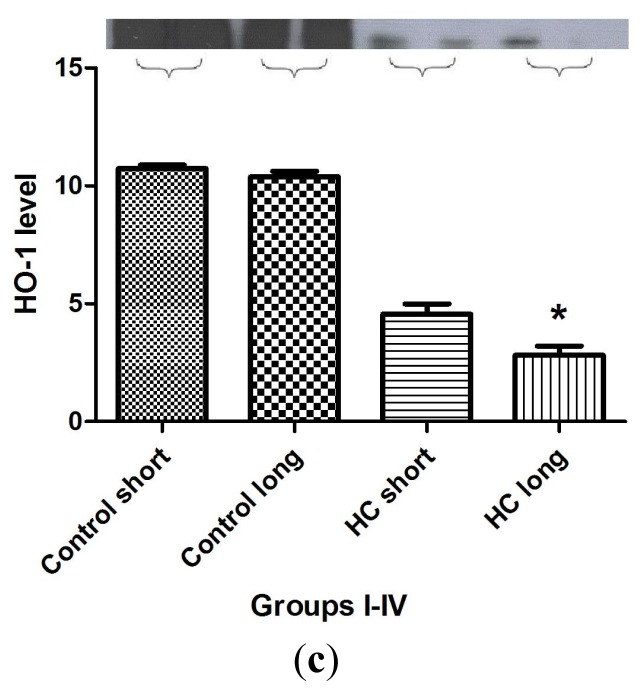
Western blot analysis for biomarkers of cardiac tissue function. Expression of COX III (7a), VEGF (7b) and HO-1 (7c) protein in left ventricular rabbit myocardium was measured in homogenized cardiac tissue samples drawn from hearts harvested from four groups of rabbits (*n* = 4 per group), administered feed containing normal chow for 12 weeks (control short); normal chow for 40 weeks (control long); 2% cholesterol during a 12-week time-course (HC short); or 2% cholesterol during a 40-week time-course (HC long). GAPDH expression level was measured as a reference protein. Western blot analyses were conducted on each tissue homogenate in triplicate, and the signal intensity of the resulting bands corresponding to proteins of interest was measured using the Scion for Windows Densitometry Image program, version Alpha 4.0.3.2 (Scion Corporation, MD, USA). Tissue content of each protein is shown in arbitrary units as the mean for each group of rabbits ± SEM. *p < 0.05 for comparison of average levels of COX III and HO-1 in myocardium of HC long animals with all other groups. # *p <* 0.05 for comparison of average levels of COX III in myocardium of HC long animals with HC short animals. (**a**) COXIII protein expression in rabbit myocardium; (**b**) VEGF protein expression in rabbit myocardium; and (**c**) heme oxygenase-1 (HO-1) protein expression in rabbit myocardium.

Figure 7 shows the outcomes of Western blot analysis for three major mediators of inflammatory signaling and cardiac homeostasis: COXIII (Figure 7a), VEGF (Figure 7b) and heme oxygenase-1 (HO-1) (Figure 7c). For these studies, left ventricular tissue from hearts freshly harvested following sacrifice of the animals was used as a source of the material used in assay for each protein of interest. Levels of COXIII enzyme in myocardial tissue from hypercholesterolemia animals administered high cholesterol feed for 40 weeks (HC long) was significantly elevated relative to the presence of the protein in hearts of animals receiving high cholesterol feed for 12 weeks (HC short) (Figure 7a) (*p <* 0.05). Interestingly, COXIII expression in cardiac tissue of HC long rabbits was observed to be significantly less than in myocardium of both groups of non-hypercholesterolemia control animals (Figure 7a) (*p <* 0.05). Evaluation of VEGF protein levels in myocardium of animals used in the present study demonstrated no apparent significant differences between levels found in non-hypercholesterolemia control rabbits and those experiencing diet-induced hypercholesterolemia (Figure 7b), nor was any significant difference in VEGF expression observed when heart tissue of HC long and HC short animals was compared (Figure 7b). By contract, when tissue expression of HO-1 was evaluated in each group, it was observed that significantly lower levels of HO-1 protein were detected in HC long heart tissue relative to hearts from the non-hypercholesterolemia control animals and, also, in comparison to HO-1 expression in HC short rabbits (Figure 7c) (*p <* 0.05).

**Figure 8:**
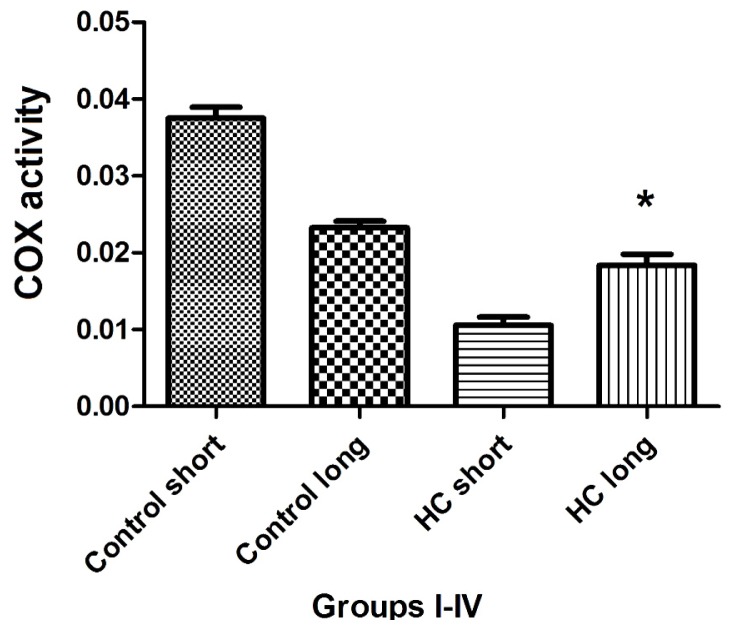
Total cytochrome *c* oxidase (COX) activity in cardiac tissue. Total COX activity in rabbit myocardium was measured in homogenized left ventricular cardiac tissue samples drawn from hearts harvested from four groups of rabbits (*n* = 4 per group), administered feed containing normal chow for 12 weeks (control short); normal chow for 40 weeks (control long); 2% cholesterol during a 12-week time-course (HC short); or 2% cholesterol during a 40-week time-course (HC long). Tissue COX activity was measured as the average decrease in absorbance wavelength of 550 nm (−ΔA_550_/min) ± SEM by mitochondrial COX-mediated conversion of reduced ferrocytochrome c to its oxidized form in mitochondria isolated from rabbit heart samples. *p < 0.05 for comparison of average COX activity in cardiac tissue of HC long animals with HC short group.

The total activity of cytochrome oxidase (COX) in left ventricular tissue of each group of animals was evaluated as the rate at which reduced ferrocytochrome c is converted to its oxidized form by homogenized rabbit heart samples. Outcomes of these experiments shown in Figure 8 demonstrate that total COX activity in heart tissues of Hypercholesterolemia animals maintained on high cholesterol diets for 40 weeks (HC long) is significantly elevated relative to the activity of the enzyme in hearts of hypercholesterolemia rabbits receiving high cholesterol chow for 12 weeks (HC short) (*p <* 0.05). Moreover, total COX activity in HC long animals was observed to be significantly lower than in non-hypercholesterolemia control animals (*p <* 0.05) (Figure 8).

Hypercholesterolemia is a disorder characterized by sustained elevation in serum cholesterol, with a resulting progressive loss of arterial flexibility and atheromatous plaque formation, leading to narrowing of critical blood vessels, particularly arteries of the heart. Typically, these plaques are physically unstable and prone to break free, releasing their components into the vasculature [25]. Plaque detritus released in this manner may block vessels supplying the heart, brain and other organs [26], resulting in tissue injury, due to lack of oxygen, called ischemia, which is compounded by extreme oxidative stress placed on tissues when and if the supply of oxygenated blood is restored (reperfusion). Ischemia and reperfusion injury are thus leading contributors to a wide range of cardiovascular pathologies, especially stroke and heart attack [27].

The present investigation evaluated the effect of diet-induced hypercholesterolemia on several critical indicators of cardiac health. Here, the investigators focused on examination of correlates of cardiovascular pathologies that provide insight into how the consequences of disruption of cardiovascular tissue homeostasis by exposure to sustained high levels of cholesterol lead to deterioration in the ability of tissue to adapt to oxidative stress. In particular, the evaluation of the degree to which elevated cholesterol affected adaptive responses, such as oxidative stress-induced changes in COX III and HO-1 activities, yields insight into how these enzymes and other metabolites, such as VEGF, may either protect against hypercholesterolemia-associated tissue damage or, in some cases, contribute to the progression of disease.

The preliminary experiments undertaken by the investigators revealed as expected that serum cholesterol levels in animals administered feed supplemented with 2% cholesterol developed a hypercholesterolemic serum cholesterol profile, with rabbits fed the high cholesterol chow for 40-week periods exhibiting significantly higher cholesterol level than those on 2% cholesterol for 12 weeks and control animals receiving normal rabbit chow (Figure 1). Furthermore, as expected, animals treated with 2% cholesterol for 40 weeks developed significantly more extensive coverage of plaque in thoracic arteries than those fed high cholesterol for 12 weeks or control rabbits (Figure 2). Likewise, significantly more extensive infarcted regions of hearts taken from rabbits treated for 40 weeks with high cholesterol chow were observed than in hearts from animals treated for 12 weeks with 2% cholesterol-supplemented feed and control animals consuming normal feed (Figure 3).

Data shown in Figure 4, which demonstrates significant reduction in the magnitude of fractional shortening (FS) and ejection fraction (EF) of the left ventricle in animals treated with 2% cholesterol for 40 weeks *versus* hearts from 12-week HC fed and normally-fed controls, yield insight into the mechanisms by which hypercholesterolemia and resulting elevation of oxidative stress and inflammatory processes may lead to cardiac insufficiency and heart failure. The design of these experiments was guided by an intriguing observation by other investigators that a pro-angiogenic drug called secoisolariciresinol diglucoside significantly protects against ischemia-reperfusion injury to hearts in a diet-induced hypercholesterolemic rat model by reduction of oxidative stress, accompanied by decreased infarct size of isolated hearts, increased HO-1 expression and significant increases in FS and EF [28]. This precedent underscores the importance of FS, EF and parameters for the assessment of the severity of hypercholesterolemia-associated cardiac insufficiency and therapeutic management of this and related cardiac disorders.

Results of experiments evaluating the effect of hypercholesterolemia on aortic flow (AF), which is a widely accepted measure of a heart’s capacity to maintain healthy oxygenation, are shown in Figure 6. As expected, the greatest reduction in cardiac AF capacity was observed in animals treated for 40 weeks on 2% cholesterol, with significantly lower flow rates recorded relative to the rates measured in control animals receiving normal chow and those fed with high cholesterol feed for 12 weeks. This outcome is expected based on the stenosis (narrowing) of critical blood vessels that occurs, due to intimal plaque buildup and inflammation-associated vasoconstriction.

The outcome of studies conducted to evaluate the effect of hypercholesterolemia on the expression of COXIII, VEGF and HO-1 are shown in Figure 7. As in previous experiments, animals exposed for the longest time periods (40 weeks) to high cholesterol diets exhibited the most significant changes. Left ventricular tissue taken from HC long (40 weeks) animals were found to contain significantly more COXIII protein than hearts from HC short (12 weeks) rabbits, but significantly less COXIII than control animals receiving normal chow (Figure 7a). The physiologic significance of this observation cannot be fully defined based on the data presented here; however, this intriguing finding may be related to the effect of elevated cholesterol on mitochondrial metabolism. A report published in 2010 demonstrates that exposure of porcine cardiac endothelium responding to interaction with oxidized LDL (which is a known feature of the hypercholesterolemic state) exhibited significant dysregulation of mitochondrial enzyme function, including COXIII [29]. Thus, hypercholesterolemic animals are more likely to experience disruption in COXIII activity; however, the specific effects shown in Figure 7 will need further analysis in order to place them in a mechanistic picture of hypercholesterolemic disruption of mitochondrial function. The lack of significant differences in the VEGF content of heart tissue from any of the groups of rabbits included in the present study is puzzling given the adverse effect on neovascularization of hypercholesterolemia observed in rats and the pivotal role played by VEGF in this process (Figure 7b) [28]. The outcome of these studies also merits further exploration in future research. Hypercholesterolemia engenders processes that greatly increase the level of oxidative stress to which cardiac tissue is subjected. Therefore it is unsurprising that significantly lower levels of HO-1 protein were detected in hearts of animals treated for 40 weeks with high cholesterol diets (Figure 7c). HO-1 expression is an adaptive response, which is upregulated by elevation in oxidative stress. Nevertheless, the protective effect of pathophysiologic increase of HO-1 activity is often overwhelmed by the intensity of a particular type of trauma to which the tissue is subject [30]; in this case, the hypercholesterolemic condition imposed by diet.

These results are intriguing in the context of observations made in a previous study by the authors, which demonstrated that serum cholesterol levels in hypercholesterolemic rabbits administered sour cherry extract in addition to 2% cholesterol-enriched feed was significantly lower than in hypercholesterolemic animals not receiving the extract [3]. Moreover, the extract-treated rabbits exhibited greatly improved cardiac function, reduced arterial atherosclerotic plaque and lower infarct size than untreated controls [3]. These effects, which positively correlated with HO-1 expression in heart tissue, raise the possibility that statin drugs, which are also HO-1 inducers, may share common mechanisms contributing to cholesterol reduction. This possibility is nevertheless complicated by findings presented in 2011 that statins do not act primarily through HO-1 induction [31]. It is therefore likely that investigations of the relationship between statin drugs and processes in which HO-1 is a participant will yield novel strategies for prevention and management of cardiovascular disease.

Total activity of cytochrome *c* oxidase (COX) was evaluated in the cardiac tissue of test animals, with outcomes of these experiments shown in Figure 8. Here, the patterns of COX enzyme activity paralleled expression of COXIII protein with respect to the status of the animals from which cardiac tissue was harvested to assay for COX protein using Western blot (Figure 7) or COX-mediated conversion of reduced ferrocytochrome c to its oxidized form (Figure 8). Left ventricular tissue taken from HC long (40 weeks) animals exhibited significantly greater ability to convert COX substrate than heart tissue from HC short (12 weeks) rabbits, but significantly less than control animals receiving normal chow (Figure 8). Although data presented here does not permit an evidence-based interpretation of the mechanistic basis for these observations, here, too, it is likely that high levels of oxidized LDL resulting from the hypercholesterolemic condition of the animals has impaired both function and expression of critical mitochondrial enzymes, including COX [29].

## 3. Experimental Section

### 3.1. Animals and Induction of Hypercholesterolemia

The experiments were carried out with adult male New Zealand rabbits with a body weight range of 2.0–2.5 kg. Animals received humane care in compliance with the “Principles of Laboratory Animal Care” formulated by the National Society for Medical Research, prepared by the National Academy of Sciences (publication No. 86 23, revised 1985). The rabbits were provided with laboratory rodent chow (normal) or chow enriched with 2.0% cholesterol (Godollo LTD, Budapest, Hungary), daily for 40 weeks *ad libitum*.

### 3.2. Serum Cholesterol Measurement

Serum cholesterol levels in the venous blood of each animal were measured using a CardioCheck serum cholesterol analyzer (Point Of Care Diagnostics, Ltd., Artarmon, New South Wales, Australia) at time-points 0 (baseline), 4, 8, 12, 32 and 40 weeks following initiation of feeding.

### 3.3. Echocardiography

Echocardiography was conducted under light anesthesia (ketamine 15 mg/kg, xylazine 3 mg/kg, intramuscular (i.m.). The chest of each rabbit was shaved, and the animal was positioned in a dorsal decubitus position. Imaging of each heart was accomplished using a VEVO 770 High-Resolution In Vivo Micro-Imaging System (FUJIFILM VisualSonics, Inc., Toronto, ON, Canada) with fundamental imaging modality. Images were stored on magneto-optical disks for off-line analysis. Parasternal long axis views were obtained and recorded to ensure that the mitral and aortic valves, as well as the apex were visualized. The exact position of the transducer was adjusted as necessary to acquire standard images. The parasternal short axis views were recorded at the mid-papillary muscle level. M-mode tracings were performed at the mid-papillary muscle level, either in parasternal long or short axis views. M-mode for visualization and quantification of wall motion in cardiovascular research was used; single line acquisition allows for the very high-temporal (1000 fps) resolution necessary for analysis of LV function. All measurements were made by a single observer, who was blind to the identity of the tracings. All measurements were averaged over three to five consecutive cardiac cycles.

Echocardiographic measurements included septal (IVSTD) and posterior (PWTD) left ventricular (LV) wall thickness in diastole, LV cavity size (end-diastolic (LVEDD) and end-systolic (LVESD) dimensions and aortic root and left atrial anteroposterior diameter. Fractional shortening was computed as follows: (LVEDD − LVESD)/LVEDD, and as global systolic function was balanced, the ejection fraction (EF) was derived as EF = (LVEDD2 − LVESD2)/LVIDD2. Left ventricular myocardial mass was calculated using LVMass (Troy) = 1.05 × [(LVEDD + posterior wall thickness at end diastole (PWTD) + IVSTD) × 3 − (LVEDD) × 3].

### 3.4. Rabbit Heart Isolation

Following the 12- and 40-week period, during which the animals were administered normal or 2% cholesterol-enriched chow, heparin (1000 IU/kg) and ketamine/xylazine (40/5 mg/kg) were injected intravenously. Next, thoracotomies were performed on each animal; the hearts were excised and placed into ice-cold perfusion buffer. The aortas were then cannulated, and hearts were perfused according to Langendorff method for a 5-minute washout period at a constant perfusion pressure equivalent to 100 cm of water (10 kPa). The perfusion medium consisted of a modified Krebs-Henseleit bicarbonate buffer: 118 mM NaCl, 4.7 mM KCl, 1.7 mM CaCl_2_, 25 mM NaHCO_3_, 0.36 mM KH_2_PO_4_, 1.2 mM MgSO_4_ and 10 mM glucose [32]. The left atrium was cannulated, and the Langendorff system was adapted and switched to isolated working hearts, as previously described in a rat model [33], adapted for rabbit heart experiments [34]. The revised procedure used a left atrial filling pressure of 17 cm (1.7 kPa) and aortic afterload pressure of 90 cm (9.0 kPa) of buffer. Aortic flow was measured by a calibrated flow meter (Gilmont Instruments, Barrington, IL, USA), and coronary flow rate was measured by a timed collection of the coronary perfusate that dripped from the heart.

Following a 10-min aerobic perfusion of the heart, cardiac function was recorded and monitored throughout the experimental period by a computer system that monitored silver electrodes and pressure transducers connected directly to the isolated hearts (ADInstruments, PowerLab, Castle Hill, Australia) in the normal control and hypercholesterolemic groups. Before ischemia, and during reperfusion, heart rate (HR), coronary flow (CF) and aortic flow (AF) rates were registered. Left ventricular developed pressure (LVDP) was also recorded by the insertion of a catheter into the left ventricle via the left atrium and mitral valve [32]. The hemodynamic parameters were registered by a computer acquisition system (ADInstruments, PowerLab, Castle Hill, Australia).

The isolation of hearts, ischemia/reperfusion and the measurements of cardiac function were conducted following 12 weeks of cholesterol-supplemented diet, and comparisons were made between the age-matched non-cholesterolemic and hypercholesterolemic groups. Cardiac function was registered before the induction of normothermic global ischemia and during reperfusion.

### 3.5. Infarct Size Determination

100 mL of 1% triphenyl tetrazolium chloride (TTC) solution in phosphate buffer (Na_2_HPO_4_ 88 mM, NaH_2_PO_4_ 1.8 mM) was administered via the side arm of the aortic cannula and, then, stored at −70 °C for later analysis. Hearts were sliced transversely [35] in a plane perpendicular to the apicobasal axis into 2–3 mm thick sections, weighted, blotted dry, placed in between microscope slides and scanned on a Hewlett-Packard Scanjet 5p single pass flatbed scanner (Hewlett-Packard, Palo Alto, CA, USA). Using NIH 1.61 image processing software (Hewlett-Packard, Palo Alto, CA, USA), each image was subjected to equivalent degrees of background subtraction, brightness and contrast enhancement for improved clarity and distinctness. The infarct area of each slice was traced, and the respective areas were calculated by pixel density analysis [36]. With the use of the NIH Image 1.61 image processing software, each digitalized image was subjected to equivalent degrees of background subtraction and brightness/contrast enhancement for improved clarity. Infarct size was expressed as a percentage ratio of the infarct zone to the risk zone (weight of the left ventricle). Here, triphenyl tetrazolium, (TTC) staining, a direct, post-sacrifice approach to imaging the infarcted areas, is used to avoid problems in clearly demonstrating infarcted zones, along with high cost intrinsic to recently-developed non-invasive imaging methodologies used with live animals [37,38].

Previously, the authors have demonstrated the elevated occurrence of cardiac infarction in rabbits with diet-induced hypercholesterolemia *versus* healthy control animals. These studies showed that systemic reduction of oxidative stress by oral treatment with sour cherry seed extract, an inducer of heme oxygenase-1, resulted in significant reduction in the infarct size observed in hearts of hypercholesterolemic rabbits treated with the extract *versus* untreated controls [3]. Occurrence of infarction observed in the present study may be accounted for by elevated arterial plaque coverage observed in hypercholesterolemic animals shown in Figure 2. These results are consistent with previous human studies demonstrating that progressive restriction of blood flow to the heart and plaque-associated inflammation increases oxidative stress on cardiac tissues and contributes to infarction [39,40].

### 3.6. Analysis of Atherosclerotic Lesions

Quantification of fatty streaks was performed with Sudan III stain. Thoracic arteries were harvested, dissected free of excess connective tissue and fat, rinsed with modified Krebs-Henseleit buffer and fixed in 10% (*v*/*v*) buffered formalin. Carotid arteries were then opened longitudinally and exposed to 5 mg/mL Sudan III in 70% (*v*/*v*) isopropanol for 15 min in a water bath at 37 °C, and the stain was differentiated with several rinses of 70% isopropanol. The arteries were then scanned, and the atherosclerotic plaque was determined [32,36].

### 3.7. Cytochrome *c* Oxidase (COX) Activity

The activity of cytochrome *c* oxidase in rabbit myocardium was measured using a colorimetric assay kit for oxidation of cytochrome c by this enzyme (Sigma-Aldrich, St. Louis, MO, USA). Briefly, mitochondria isolated from freshly harvested heart muscle using the MITOISO1 kit (Sigma-Aldrich, St. Louis, MO, USA) were treated with dithiothreitol to reduce cytochrome c, followed by COX-mediated reoxidation of the molecule. COX activity at room temperature (~22 °C) in each sample was measured as a decrease in absorbance of ferrocytochrome c, at an absorbance wavelength of 550 nm (UV Helios Alpha S2 spectrophotometer, (UNICAM, Budapest, Hungary) in its conversion from a reduced to oxidized state. The COX activity in any particular sample was reported as (−ΔA_550_/min).

### 3.8. Western Blot Analysis

Total protein (100 μg) in the Clontech Extraction buffer was added to an equal volume of sodium dodecyl sulfate (SDS) buffer and boiled for 10 min before being separated on 12% SDS polyacrylamide gels in a running buffer (25 mM Tris, 192 mM glycine, 0.1% (*w*/*v*) SDS, pH 8.3) at 120 V. The Precision plus Protein Kaleidoscope standards (10 L) (Bio-Rad Laboratories, Hercules, CA, USA) were used as molecular-weight standards. The gel was transferred onto a nitrocellulose membrane (Bio-Rad Laboratories, Hercules, CA, USA) at 100 V and left for 1 h in a transfer buffer (25 mM Tris base, 192 mM glycine, 20% (*v*/*v*) methanol, pH 8.3). After blocking the membranes for 1 h in a Tris-buffered saline (TBS-T) (50 mM Tris, pH 7.5, 150 mM NaCl) containing 0.1% (*v*/*v*) Tween-20 and 5% (*w*/*v*) nonfat dry milk, blots were incubated overnight at 4 °C with the primary antibody (HO-1, VEGF, COX III, COX IV, GAPDH). Membranes were washed 3 times in TBS-T before being incubated for 1 h with horseradish peroxide (HRP)-conjugated secondary antibody diluted 1:2000 in TBS-T and 1% (*w*/*v*) nonfat dry milk. Detection was made by autoradiography for variable lengths of time with medical X-ray film (Agfa-Gevaert N.V., Mortsel, Belgium). GAPDH (cytoplasm) and COX IV (mitochondrial) were used as the loading controls (Sigma-Aldrich, St. Louis, MO, USA). Quantitative analysis of scanned Western blots to estimate the levels of HO-1, VEGF, COX III, COX IV and GAPDH protein in each sample were calculated using the Scion for Windows Densitometry Image program, version Alpha 4.0.3.2 (Scion Corporation, Maryland, MD, USA). Signal intensity for bands corresponding to each protein of interest was estimated and reported in arbitrary units ± SEM. For these experiments, the Scion Densitometry Image program was selected as the most cost-effective for evaluation of selected proteins. The product is public domain software derived from an imaging program used routinely by the United States National Institutes of Health (NIH). It was selected for the present study based on its diverse range of application and reliability.

### 3.9. Statistics

Results are expressed as the mean ± SEM (*n =* 4 in each group). One-way analysis of variance was first carried out to test any differences between the mean values of different groups. If differences were established, the results between two groups were compared by the Tukey test. Results were considered to be significant if *p <* 0.05.

## 4. Conclusions

The major outcomes of the present study are summarized in Table 2. Experiments conducted by the authors establish clear correlation between the length of time an animal’s cardiovascular system is exposed to the hypercholesterolemic state and dysregulation of homeostatic processes critical to cardiac health. The findings reported here further provide a frame of reference for characterization of the mechanisms of cardiac disease associated with hypercholesterolemia.

## Figures and Tables

**Table 1:** Values for M-mode and 2D echocardiographic variables in New Zealand white rabbits.

Control Short					

Cardiac Parameters	Mean ± SD	Minimum	Maximum	Reference range *	Coefficient of variation (%)
FS (%)	34.97 ± 5.085	25.77	44.44	17.63 to 40.16	14.54
EF (%)	60.08 ± 6.712	47.13	71.76	42.42 to 75.65	11.17
IVSd (mm)	2.81 ± 1.063	1.92	4.99	2.11 to 2.82	37.81
IVSs (mm)	4.308 ± 0.7011	3.23	5.63	2.98 to 5.92	16.27
LVIDd (mm)	17.07 ± 1.574	14.84	19.38	13.59 to 17.89	9.22
LVIDs (mm)	11.09 ± 1.173	8.95	12.93	8.51 to 13.63	10.57
LVPWd (mm)	3.102 ± 1.003	0.81	4.61	1.89 to 3.63	32.33
LVPWs (mm)	4.815 ± 1.173	2.93	6.42	2.50 to 5.43	24.35
LV mass (mg)	7532 ± 1707	3232	9911	na	22.67

**Control Long**					

**Cardiac Parameters**	**Mean ± SD**	**Minimum**	**Maximum**	Reference range*	**Coefficient of variation (%)**

FS (%)	33.21 ± 4.035	28.07	40.61	17.63 to 40.16	12.15
EF (%)	57.63 ± 5.635	50.03	68.15	42.42 to 75.65	9.78
IVSd (mm)	2.474 ± 0.5083	1.86	3.89	2.11 to 2.82	20.55
IVSs (mm)	4.083 ± 0.7418	3.05	5.47	2.98 to 5.92	18.17
LVIDd (mm)	19.09 ± 3.852	12.83	25.95	13.59 to 17.89	20.18
LVIDs (mm)	12.84 ± 3.059	7.62	18.49	8.51 to 13.63	23.83
LVPWd (mm)	3.177 ± 1.026	0.6	4.96	1.89 to 3.63	32.3
LVPWs (mm)	4.574 ± 1.461	2.91	8.4	2.50 to 5.43	31.93
LV mass (mg)	8876 ± 2658	4968	15,539	na	29.95

**HC Short**					

**Cardiac Parameters**	**Mean ± SD**	**Minimum**	**Maximum**	Reference range*	**Coefficient of variation (%)**

FS (%)	31.67 ± 6.460	24.2	45.35	na	20.4
EF (%)	55.38 ± 8.707	44.75	73.35	na	15.72
IVSd (mm)	2.389 ± 0.5772	1.39	3.45	na	24.16
IVSs (mm)	3.718 ± 1.011	1.91	5.92	na	27.19
LVIDd (mm)	17.58 ± 2.109	14.11	20.5	na	11.99
LVIDs (mm)	12.04 ± 2.065	7.71	14.22	na	17.14
LVPWd (mm)	3.2 ± 1.181	1.56	5.57	na	36.9
LVPWs (mm)	4.279 ± 1.164	2.71	6.86	na	27.21
LV mass (mg)	7571 ± 2545	3312	10,738	na	33.62

**HC Long**					

**Cardiac Parameters**	**Mean ± SD**	**Minimum**	**Maximum**	Reference range*	**Coefficient of variation (%)**

FS (%)	20.44 ± 2.946	17.58	24.55	na	14.41
EF (%)	38.45 ± 4.798	33.6	45.22	na	12.48
IVSd (mm)	2.985 ± 0.7013	2.17	4.3	na	23.49
IVSs (mm)	3.837 ± 0.8885	2.32	5.24	na	23.15
LVIDd (mm)	18.27 ± 1.938	15.62	21.61	na	10.6
LVIDs (mm)	14.52 ± 1.477	12.86	17.35	na	10.17
LVPWd (mm)	3.392 ± 1.237	0.86	5.18	na	36.46
LVPWs (mm)	4.585 ± 1.444	1.9	6.76	na	31.5
LV mass (mg)	9639 ± 3313	2838	14,147	na	34.37

FS, fractional shortening of left ventricle; EF, ejection fraction, IVSd, interventricular wall thickness in diastole; IVSs, interventricular wall thickness in systole; LVIDd, left ventricular internal diameter at end-diastole; LVIDs, left ventricular internal diameter at end-systole; LVPWd, left ventricular posterior (free) wall thickness in diastole; LVPWs, left ventricular posterior (free) wall thickness in systole; LV mass, mass of left ventricle; (*n =* 4 in each group);

* reference range for healthy New Zealand white rabbits [23]. Standardized reference ranges for HC short and HC long values in a hypercholesteremic rabbit model maintained under the conditions described in the present report were not found in public domain literature sources and accordingly are denoted “na” below in Table 1.

**Table 2:** A summary of the experiments conducted in this study and their major outcomes.

In-text figure	Description of experiments	Major outcomes
1	Serum cholesterol evaluated in rabbits treated for 12- and 40-week periods with 2% cholesterol-enriched feed.	Significantly higher cholesterol levels in animals receiving cholesterol-supplemented feed.
2	Plaque coverage evaluated in thoracic arteries.	Significantly elevated plaque coverage in blood vessels of hypercholesterolemia rabbits *versus* healthy controls and short-term hypercholesterolemia.
3	Extent of infarcted zones evaluated in hearts of subject animals.	Non-hypercholesterolemia: *No infarction*. 12-week high cholesterol: *No*/*Minimal*. 40-week high cholesterol: *Extensive*.
4,5	Echocardiographic measurements made in hearts of subject animals.	Significant reduction in left ventricular fractional shortening (FS) and ejection fraction (EF) in the hypercholesterolemia long group.
6	Aortic flow measurements were made in hearts of subject animals.	Significant reduction in aortic flow of rabbits treated long-term with high cholesterol diets.
7	Western blot analysis of left ventricular tissue for mediators of cardiovascular homeostasis.	Content of major mediators in hearts of hypercholesterolemia *versus* control animals: COXIII, 12 week: significantly decreased in HC short group *versus* normal COXIII, 40 week: significantly elevated in HC long group *versus* HC short group. VEGF: no significant differences. HO-1, 12-week: HC short group has significantly lower expression compared to normal. HO-1, 40-week: significantly lower. HC long showed further decreased expression.
8	Total cytochrome *c* oxidase (COX) activity was measured in left ventricular tissue of subject animals.	12 week: significantly decreased in HC short group *versus* normal. 40 week: significantly elevated in HC long group *versus* HC short group.
